# Pharmacogenomics and Pharmacometabolomics in Precision Tramadol Prescribing for Enhanced Pain Management: Evidence from QBB and EMR Data

**DOI:** 10.3390/ph18070971

**Published:** 2025-06-27

**Authors:** Dhoha Dhieb, Najeha Anwardeen, Dinesh Velayutham, Mohamed A. Elrayess, Puthen Veettil Jithesh, Kholoud Bastaki

**Affiliations:** 1 Pharmaceutical Sciences Department, College of Pharmacy, QU Health, Qatar University, Doha P.O. Box 2713, Qatar; dhoha.dhieb@qu.edu.qa; 2 Biomedical Research Center, QU Health, Qatar University, Doha P.O. Box 2713, Qatar; n.anwardeen@qu.edu.qa (N.A.); m.elrayess@qu.edu.qa (M.A.E.); 3 College of Health & Life Sciences, Hamad Bin Khalifa University, Doha P.O. Box 34110, Qatar; dineshvelayutham@qf.org.qa (D.V.); jveettil@hbku.edu.qa (P.V.J.)

**Keywords:** pain management, tramadol, pharmacometabolomics, pharmacogenomics

## Abstract

**Background/Objectives**: Tramadol is an opioid frequently prescribed for moderate to severe pain and has seen a global increase in use. This presents numerous challenges in clinical management. This study aims to elucidate metabolic signatures associated with tramadol consumption, enhancing predictive capabilities for therapeutic outcomes and optimizing patient-specific treatment plans. **Methods**: Data were obtained from the Qatar Biobank (QBB), focusing on pharmacogenomic variants associated with tramadol use and prescription trends. A cohort of 27 individuals who were administered daily tramadol doses between 100 and 400 mg with available metabolomic profiles were selected. The pharmacokinetics of tramadol were evaluated in relation to specific CYP2D6 genetic variants. Comparative pharmacometabolomic profiles were generated for tramadol users versus a control group of 54 non-users. Additionally, prescription data encompassing tramadol formulations were collected from the electronic medical records (EMR) system of the major public hospital network in Qatar (Hamad Medical Corporation) to discern prescribing patterns. **Results**: From January 2019 to December 2022, tramadol prescriptions varied, with chronic pain as the primary indication, followed by acute pain. Pharmacogenomic analysis indicated that CYP2D6 allele variations significantly impacted tramadol and O-desmethyltramadol glucuronide levels, notably in ‘normal metabolizers’. Metabolomic analysis revealed distinct metabolic profiles in tramadol users, with significant variations in phosphatidylcholine, histidine, and lysine pathways compared to controls, highlighting tramadol’s unique biochemical impacts. **Conclusions**: This study underscores the importance of integrating genetic and omics-based approaches to enhance tramadol’s efficacy and safety. These findings support personalized pain management strategies, enhancing treatment outcomes for both chronic and acute pain.

## 1. Introduction

Pain is one of the most common reasons that patients seek medical attention, and it is one of the leading causes of visits to primary health care providers [1,2]. Chronic pain, a major global health issue, affects nearly 100 million individuals in the US alone, with an approximate annual economic burden of USD 635 billion due to lost wages and healthcare expenses [3,4]. When pain management incorporates multiple strategies that include pharmacological and non-pharmacological interventions, it becomes complex because different types of hurts (nociceptive, neuropathic, psychogenic) overlap among certain syndromes [5]. Individuals presenting multiple chronic pains complicate clinical definitions, whereas gaps exist regarding the most effective treatment plans [6]. Pharmacological intervention depends on pain type: nociceptive (acetaminophen or nonsteroidal anti-inflammatory drugs) or neuropathic (antidepressants or antiepileptic medications). Severe acute forms of cancer or postoperative discomforts often necessitate opioid therapy despite controversies surrounding their use for chronic non-cancer ailments [7,8].

A key player in pain management is tramadol, a synthetic opioid with a unique dual analgesic effect [9]. It acts as a mixed mechanism opioid by centrally activating the μ-opioid receptor, while inhibiting norepinephrine and serotonin reuptake [10,11]. For that reason, it is prescribed for treating pain, which ranges from moderate to severe chronic pain to conditions, like chemotherapy-induced neuropathy. It is occasionally used as a secondary option for fibromyalgia patients who do not respond to initial therapies. However, its effectiveness for various chronic pains, including neuropathic pain, remains uncertain [5]. In the context of Qatar, tramadol is available in three formulations: immediate release, extended release, and oral drops. Besides its primary therapeutic use for analgesic purposes, it is also employed off-label to treat conditions, such as premature ejaculation and restless leg syndrome [5]. Notably, while tramadol offers a potential option for cancer pain relief when compared with placebos or active controls, clinical evidence suggests its efficacy may be outperformed by conventional mu agonist opioids, like morphine [12]. Tramadol hydrochloride is structurally analogous to morphine and codeine, comprising two enantiomers: (+)-tramadol and (−)-tramadol [13,14]. Administered as a racemic mixture, tramadol exhibits variable pharmacokinetic properties across these enantiomers [15]. This FDA-approved medication, classified as a class IV drug since 2014, is designed for moderate to severe pain relief [16,17], particularly when other non-opioid medications have proven ineffective [5]. Despite its global prescription and efficacy, there is potential for misuse among opioid-dependent subjects [18] and risk of severe adverse drug effects upon overdose, such as seizures, respiratory depression, coma, nausea, hypotension, vomiting, or serotonin syndrome [19,20,21]. It is prescribed cautiously due to its potential for abuse and addiction [16,17]. Tramadol is rapidly absorbed, distributed, and undergoes significant first-pass metabolism (around 20–30% of administered dose) with a bioavailability of 68–84% [13,14,19,22]. Metabolization primarily occurs in the liver [19,22], catalyzed by the cytochrome P450 (CYP) enzymes CYP2D6 and CYP3A4-CYP2B6, producing the active metabolites *O*-desmethyltramadol (M1) and *N*-desmethyl-tramadol (M2), respectively [15,23,24,25]. M1, which has a markedly higher affinity for μ-opioid receptors compared to tramadol, plays a major role in opioid activity and pain relief [22,26]. Pharmacokinetics and pharmacodynamics are significantly influenced by the genetic variability of CYP2D6. Over 150 genetic variants of CYP2D6 affecting the enzyme’s metabolic activity have been defined [27,28]. These genetic variations are categorized into four metabolizer phenotypes: poor metabolizers (PMs), intermediate metabolizers (IMs), normal metabolizers (NMs), and ultra-rapid metabolizers (UMs) [29,30], associated with different drug responses and toxicity profiles [31].

Recent reports indicate a fourfold increase in adverse events related to tramadol intake from 2013 to 2018, according to the WHO Global Database. These events often involve incorrect dosages, leading to overdose risks, primarily expressed through symptoms, like sedation and respiratory depression, and are typically responsive to naloxone in severe cases [32]. Long-term use of tramadol is associated with heightened co-morbidity risks, including anxiety, depression, and obsessive-compulsive episodes, but not psychotic symptoms, underscoring concerns about dependency and misuse, promoting physical dependencies [32,33]. Moreover, there is a concerning increase in tramadol misuse, particularly among younger individuals, driven by its easy availability, low cost, and misconceptions about its safety as a prescription drug. This trend is underscored by alarming statistics, including over 19,000 tramadol-related deaths in the United States [34,35]. The complexity of tramadol response, shaped by genetic, metabolic, and clinical factors, highlights the need for advanced research strategies to identify robust predictors of efficacy and safety. Recent advances in molecular research have substantially expanded our understanding of biological processes, disease mechanisms, and treatment response. Integrative molecular approaches, such as metabolomics and pharmacogenomics, enable comprehensive characterization that transcends traditional biomarker studies. For instance, cutting-edge methods in molecular representation learning, including multi-view neural networks, have greatly improved the prediction and modeling of molecular properties, facilitating artificial intelligence-driven drug discovery and personalized medicine [36]. Likewise, precision studies on the function and conservation of specific RNAs, coupled with functional genomics and pathway enrichment analyses, continue to reveal critical regulatory axes and signaling networks relevant to complex phenotypes [37]. Such studies underscore the value of integrating high-dimensional molecular data to uncover novel mechanisms, identify therapeutic targets, and guide individualized clinical decision-making. In the context of tramadol, pharmacogenomics (PGx) and pharmacometabolomics (PMx) offer considerable promise for improving the prediction of individual safety and efficacy profiles. Numerous studies have established a correlation between PGx and clinical outcomes in pain management [38,39], while others have illustrated beneficial outcomes following PGx-guided interventions in various medical conditions [40]. Current clinical pharmacogenomic guidelines provide specific recommendations for tramadol, underscoring the utility of PGx in clinical practice [41]. Nevertheless, clinical implementation can be challenging, especially in complex disorders, such as mental illnesses, including managing subtypes of chronic pain conditions [42,43]. Pharmacometabolomics provides complementary insights by analyzing molecular changes that influence phenotypic expression, bridging knowledge gaps that arise from diverse factors, such as diet and psychological components, among others. The integration of PGx approaches alongside metabolomic insights may contribute towards better diagnosis while also providing more comprehensive identification protocols discerning underlying regulatory networks governing patient responses against interventions. Our research is primarily focused on determining key aspects related to the pharmacokinetics of tramadol. A thorough genetic and metabolomic analysis is performed in this study to identify distinct metabolic signatures, which can have significant implications on personalized consumption tendencies. Our study highlights the importance of precise medicinal principles in improving current prescription practices by analyzing genetic responses associated with tramadol. Exploring metabolic signatures can lead to a greater understanding of patient-specific response outcomes and increased safety measures in clinical practice.

## 2. Results

### 2.1. Prescription Trends of Tramadol in Qatar

The analysis of prescribing trends for tramadol’s different formulations from January 2019 to December 2022 revealed unique patterns. This includes 50 mg tramadol capsules, 100 mg sustained-release tramadol tablets, and 100 mg/mL tramadol oral drops. The trend for the capsule variant was characterized by significant volatility with sharp fluctuations, culminating in a peak between July 2022 and December 2022, indicating dynamic demand. In contrast, the 100 mg sustained-release tablets exhibited a gradual increase, with a pronounced peak in mid-2020, followed by a notable decline starting in June 2022, suggesting changes in prescribing patterns aligned with patient needs. Intriguingly, the prescription volume for the 100 mg/mL oral drops demonstrated moderate yet fluctuating demand, contrary to a completely stable trend, suggesting more nuanced dynamics in usage than previously thought. These patterns highlight the evolving nature of therapeutic approaches within healthcare frameworks during our study timeline (refer to Figures S1 and S2).

In addition to formulation-specific patterns, demographic analyses drawn from Hamad records indicate that overall annual tramadol consumption remained relatively stable, with 1400–1600 individuals using tramadol per year. Males comprised just over half of the consumers throughout the four-year period. Age distribution data revealed that individuals aged 60 years and above represented the largest user group, consistently accounting for about one-third of cases annually. The 50–59 and 40–49 age groups also contributed notable proportions, collectively constituting a significant segment of tramadol users, while use in those under 20 years was minimal, making up less than 2% each year. These findings highlight that tramadol consumption in Qatar during this period predominantly affected older adults, with age and gender distributions remaining stable over time. Our investigation revealed that chronic pain management in Qatar remained the main indication for tramadol prescriptions, followed by acute pain, indicating its significant role in temporary relief from acute discomfort. However, analgesic applications, such as relief from cancer-induced distress, neuropathic disorders, and the off-label use for psychotropic conditions, maintain a comparable prescription rate and sequentially follow these primary usage categories during the study period. Analysis of clinical indications revealed that prescribing patterns for chronic and acute pain closely paralleled one another, with no significant divergence between these groups. Notably, one distinct surge in tramadol utilization was observed, in July 2020, the timing of which may coincide with COVID-19 pandemic-related restrictions. The demographic and prescribing trends established here provide a valuable reference for future, more comprehensive analyses of tramadol utilization patterns in Qatar.

### 2.2. General Characteristics of Participants

In the studied cohort, 27 subjects were discerned as actively consuming tramadol at either a dosage of 50 mg or 100 mg with an upper threshold dose of 400 mg. The median age was established at approximately 35.0 ± 13.89 years, comprising both genders, with males accounting for 10 and females constituting the remaining 17 individuals. The subject’s metabolism capability relating to CYP2D6 enzymatic activity was categorized as follows: ‘poor metabolizer’ (*n* = 1), ‘intermediate metabolizers’ (*n* = 8), ‘normal metabolizers’ (*n* = 17), and ‘ultra-rapid metabolizers’ (*n* = 1) (Refer to Table 1, Tables S1 and S2). Out of the total participants under tramadol usage investigation, there were distinctions between non-diabetic subjects (25) compared to their diabetic counterparts, numbering two individuals. The control group consisted of 54 subjects, all confirmed not to be taking tramadol, including 24 individuals who were not on any medication. This cohort included 46 non-diabetics and 8 diabetics. Comprehensive clinical characteristics related to all the subjects are cataloged in Table 1.

### 2.3. Pharmacogenomic Variation

Comprehensive WGS-based pharmacogenomic profiling of the study cohort demonstrated substantial allelic and phenotypic diversity in genes implicated in tramadol pharmacokinetics and pharmacodynamics. CYP2D6 genotype-derived metabolizer status, based on CPIC guidelines, showed 3.7% poor metabolizers (PM), 29.6% intermediate metabolizers (IM), 63.0% normal metabolizers (NM), and 3.7% ultrarapid metabolizers (UM) (Supplementary Table S1). Additional pharmacogenes were also profiled. For CYP3A4, predicted activity, inferred based on DPWG guidelines, was predominantly normal (81.5%), with intermediate activity detected in 14.8% of samples and 3.7% indeterminate. Among CYP2B6 genotypes, 55.6% were normal, 37% intermediate, and 3.7% each poor or indeterminate. Pharmacodynamic variation was notable in OPRM1, with the A118G (rs1799971) variant observed in 40.7% (AG/GG genotypes), previously associated with decreased receptor function and altered analgesic response (PharmGKB level 3). The upstream noncoding OPRM1 variant rs62436463 showed a high prevalence of the GG genotype (74.1%), linked to increased risk for tramadol-related adverse events. For ABCB1, the C3435T (rs1045642) and C1236T (rs1128503) variants showed similar genotype distributions, with the AA genotype present in 25.9% of individuals for each variant, consistent with associations in the literature suggesting increased tramadol exposure and altered pharmacokinetics (PharmGKB level 3). A detailed breakdown of all pharmacogenetic variants and corresponding clinical evidence levels is provided in Supplementary Table S1. Only CYP2D6 is currently recognized by CPIC and PharmGKB as having established clinical actionability (level 1A) for tramadol prescribing. The other pharmacogenes analyzed (CYP3A4, CYP2B6, OPRM1, and ABCB1) are considered informative markers, as evidence supporting their clinical utility remains limited or emerging.

### 2.4. Variability in Tramadol Metabolism: Correlation with CYP2D6 Activity and Metabolic Status

The thorough characterization exposed a significant range in both tramadol as well O-desmethyltramadol_glucuronide concentration among those earmarked as normal metabolizers, thereby indicating a substantial inherent variability even within this cohort category. This observed variance is critical from a safety perspective largely due to its implications wherein those designated with normal metabolic rate may not uniformly exhibit similar drug response or equivalent degree of drug-toxicity risks devoid any potential dangers. Some instances revealed exceptionally high levels among certain normal-metabolizers, which further highlight plausible toxicity risk factors due to the elevated presence of tramadol and its related derivative components. This observation may indicate a lack of efficacy and subsequent dose increases, thus signaling cautionary aspects requiring attention. This outcome illustrates considerable complexities entailed in the process involving tramadol metabolism, necessitating personalized vigilant monitoring measures accounting for specific drug concentrations so as to effectively curtail adverse medical incidents despite presumable regular metabolic status synonymous with these patients. Additional insights can be referred to in Tables S2 and S3.

### 2.5. Multivariate Analysis of Differential Metabolites

**Figure 1:**
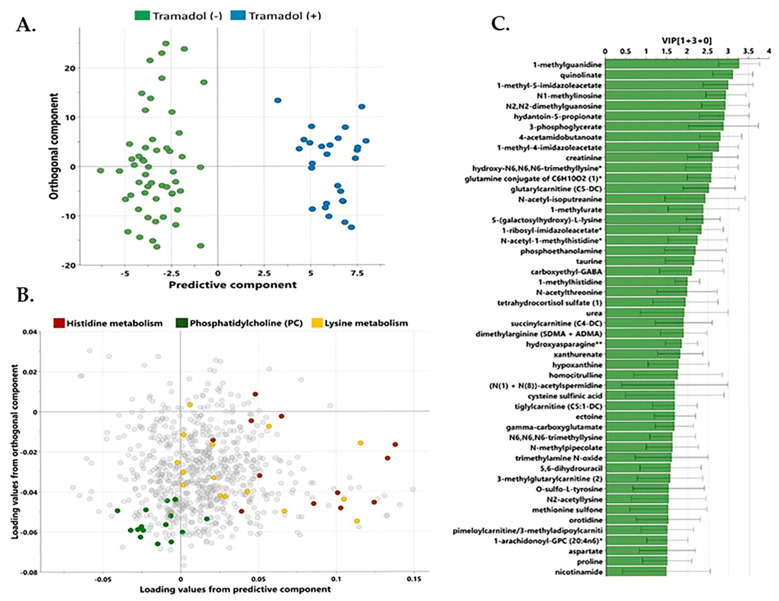
(**A**) Score plot from OPLS-DA between tramadol-positive and tramadol-negative individuals (R2Y = 93.8%, Q2 = 48.8%). (**B**) Corresponding loading plot showing the enriched pathways between tramadol-positive and tramadol-negative individuals. Each point represents an individual subject. (**C**) VIP list of metabolites from OPLS-DA analysis. Asterisk (*) next to a metabolite indicates a compound that has not been officially confirmed based on a reference standard, but Metabolon is confident in its identity; Double asterisks (**) next a metabolite indicates a compound for which a standard is not available, but Metabolon is reasonably confident in its identity or the information provided.

Utilizing a non-targeted metabolomics approach, metabolic signatures were explored in 27 patients on tramadol therapy. OPLS-DA served as the primary tool for identifying prominent components distinguishing between tramadol-positive and tramadol-negative subjects, depicted in Figure 1. The OPLS-DA exhibited one predictive and two orthogonal components. The differentiating component alone accounted for an impressive 93.8% variance between the tramadol-positive and -negative group. Figure 1 shows the list of metabolites with VIP ≥ 1.5.

### 2.6. Univariate Analysis of Differential Metabolites

**Figure 2:**
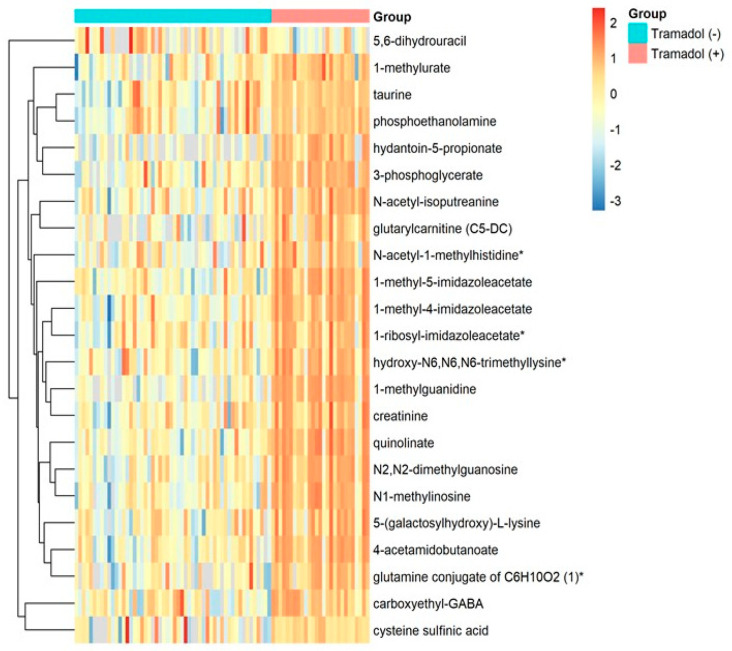
Heatmap representing the metabolites with significantly different levels (FDR ≤ 0.05) between tramadol-positive and tramadol-negative participants. Asterisk (*) next to a metabolite indicates a compound that has not been officially confirmed based on a reference standard, but Metabolon is confident in its identity.

The linear model analysis delineated significant contrasts (FDR ≤ 0.05) between the two investigated cohorts, as elaborated in Table 2. Notable alterations were evident in metabolites, such as 1-methylguanidine, quinolinate, *N*2,*N*2-dimethylguanosine, and *N*1-methylinosine, among others. Intriguing variations also surfaced within microbiota-related metabolites, encompassing 1-methyl-5-imidazoleacetate, 1-methyl-4-imidazoleacetate, and hydantoin-5-propionate. Similarly, other metabolite discrepancies, like glutamine conjugates of C6H10O2 (1), hydroxy-*N*6,*N*6,*N*6-trimethyllysine*, taurine, and phosphoethanolamine, are marked as well. A plethora of differentiating markers, including carboxyethyl-GABA and cysteine sulfinic acid, have been further identified, distinguishing between the two groups (Table 2 and Figure 2).

### 2.7. Correlation of Clinical Parameters with Identified Metabolites

**Figure 3:**
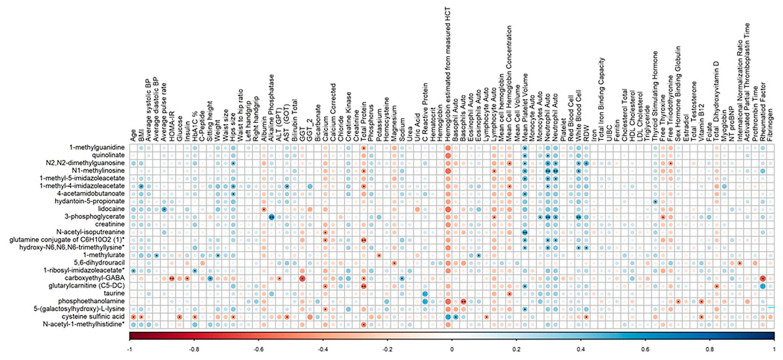
Heatmap of Spearman’s correlation between the significantly associated metabolites and clinical parameters in tramadol users. (**/*) signifies *p*-value (<0.01/<0.05), respectively. Asterisk (*) next to a metabolite indicates a compound that has not been officially confirmed based on a reference standard, but Metabolon is confident in its identity.

Spearman’s correlation analysis revealed significant associations between various clinical parameters and metabolites in tramadol users, pinpointing key metabolic pathways likely impacted by tramadol consumption. Notable findings include a robust negative correlation between insulin resistance, as indicated by HOMA-IR, and carboxyethyl-GABA (ρ = −0.506, *p*-value = 0.007), which highlights a potential interaction between insulin resistance and GABAergic metabolism. Additionally, liver function, as measured by gamma-glutamyl transferase (GGT), also exhibited a strong negative correlation with carboxyethyl-GABA (ρ = −0.620, *p* = 0.031), indicating the liver’s critical role in tramadol’s metabolic processes. Other correlations covered interactions with cardiovascular markers, metabolic and hormonal indicators, and immune response elements, all suggesting intricate biophysiological layers affected by tramadol administration (Figure 3 and Tables S4 and S5).

### 2.8. Functional Enrichment Investigation

**Figure 4:**
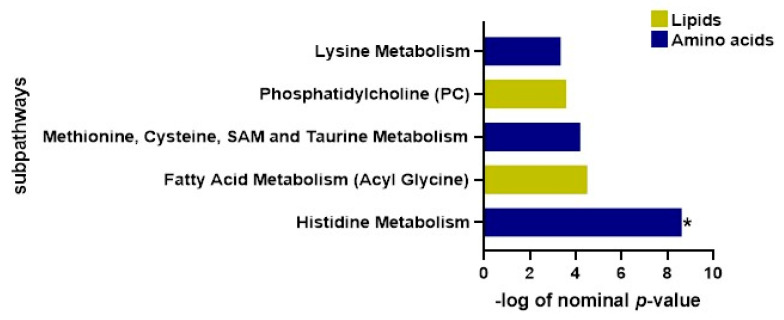
Bar plot of functional enrichment analysis results based on metabolite ranks by *p*-value using Fisher’s exact test. Asterisk (*) indicates statistical significance after correction for multiple testing (FDR < 0.05).

The results acquired from functional enrichment assessments (Figure 4 and Figure S3 and Table 3) illuminate considerable distinctions in metabolic sub-pathways, which include lysine metabolism along with phosphatidylcholine (PC), methionine metabolism, cysteine-, SAM-, taurine-focused metabolism processes, and fatty acid- and histidine-driven metabolism systems. Figure 4 illustrates these findings using a bar diagram to effectively showcase dominant metabolite entities.

## 3. Discussion

Tramadol is a widely used analgesic with central nervous system effects, making it a premier prescription choice globally for the management of moderate to severe pain. Despite its widespread use in Qatar, there is a significant lack of data on the clinical indications, prescribing patterns, and long-term effects associated with tramadol use. These knowledge gaps are significant, as they can inform policy decisions regarding future regulations surrounding tramadol use, thus upholding public health safety integrity and ensuring accessible and effective pain management strategies. Interestingly, our findings highlighted the extensive usage of tramadol predominant in chronic pain management, which can increase the risk of long-term use and potential dependency due to ongoing patient discomfort. Tramadol’s addictive potential has become a major public health concern in the last two decades, particularly in Egypt and the Middle East [44,45]. Other regions across Europe and Asia also admitted of instances of abuse of tramadol as a substance with dependence potential [46,47,48]. Patients value this substance for its pain relief, stimulative and antidepressant properties, and as a remedy for premature ejaculation [16,49,50,51]. Neurological complications found to be associated with the long-term utilization included seizures, serotonin syndrome, Alzheimer’s disease, and Parkinson’s [52]. Several factors could possibly provoke prolonged tramadol use; these include the success rate of the treatment comparative cost against the other available pharma remedies. Analyzing prescription patterns helps identify factors involved in opioid prescriptions and provides insights into long-term medication use in chronically exposed patients [53]. Some serious side effects included angioedema, increased effects of anticoagulants, hypoglycemia, and serotonin toxicity [54,55,56,57,58]. Some literature revealed that opioid abuse may lead to structural changes and apoptosis of neurons [59,60]. Zhuo pointed out that chronic exposure to tramadol could induce toxic effects on the neurotransmitters of zebrafish [61]. Mohamed et al. [55] reported that chronic exposure to tramadol induced oxidative damage, inflammation, and apoptosis on the cerebrum of rats [55]. Chronic tramadol use treatment may provoke the production of reactive oxygen species (ROS), contributing to toxicity [61,62,63]. A key observation in our study was high tramadol concentrations in patients with normal metabolizers. This finding might be attributed to the phenomenon known as phenoconversion, in which actual metabolic activity differs from genetic predictions as a result of various non-genetic influences [64,65]. Phenoconversion underscores the complex interplay between both genetic predispositions and environmental influences in determining drug response dynamics. In this context, multiple non-genetic elements, such as diet, concomitant medication usage, or physiological states could potentially manipulate enzymatic activity levels contrarily impacting expected metabolic efficacy, thereby enhancing tramadol concentrations unwittingly [66]. These findings suggest the need for precision medicine strategies that consider both genetic markers along with relevant contextual variables that determine actual metabolic responses for fine-tuning therapeutic strategies linked with opioid use, like tramadol. Phenoconversion can result from the simultaneous utilization of CYP450 inhibitors, aging, cancer, and inflammation, causing shifts towards lower metabolism phenotypes, while CYP450 inducers or smoking, alcohol use, pregnancy, and vitamin D exposure can increase metabolism [67]. Phenoconversion has been increasingly recognized as a clinically relevant challenge, with recent studies showing it to be prevalent in patients exposed to CYP2D6 inhibitors or other modulators and demonstrably altering analgesic efficacy and safety profiles [38,66,68]. These findings underscore the limitations of genotype-based precision prescribing in isolation and support recent calls for the integration of real-time metabolic phenotyping, such as metabolite profiling or enzyme activity assays, into clinical decision-making, especially for drugs with narrow therapeutic windows or complex metabolism, like tramadol. Clinical decision tools for assessing phenoconversion risk are now emerging and may facilitate the implementation of combined genotyping and phenotyping approaches in pain management [69]. Thus, clinicians should not rely solely on pharmacogenomic predictions but should also consider potential phenoconversion, particularly in the context of polypharmacy or comorbidities, when tailoring tramadol therapy for individual patients. While studies do illustrate discrepancies between genotypic–phenotypic outcomes, the clinical impact of these variations remains uncertain, underscoring the need for further research into the complex regulation of cytochrome P450 metabolism. On the other hand, we performed an intensive pharmacometabolomic study. The biggest concern of the research was therefore to disclose the complexity of the perturbation that takes place within the metabolism of tramadol exposure. The resulting metabolomic profile indicated significant differences between control groups and those exposed to tramadol. Creatine popped up as a vital biomarker with respect to kidney injury in tramadol subjects [70]. Data related to quinolinate, metabolically active in the metabolism of nicotinate and nicotinamide, showed obvious changes related to inflammation and signs of oxidative damage [14,71]. These results were intrinsically related to our KEGG pathway enrichment analysis findings, which included wide metabolites acting over different pathways, such as histidine metabolism, lysine metabolism, phosphatidylcholine (PC) metabolism, fatty acid metabolism (acyl glycine), and methionine/cysteine/S-adenosylmethionine (SAM)/taurine metabolism. These alterations are consistent with tramadol’s dual mechanism of μ-opioid receptor agonism and inhibition of monoamine reuptake, which provoke widespread neurochemical and metabolic shifts [14,72]. The perturbation of histidine metabolism may reflect compensatory histaminergic modulation, affecting arousal, nociception, and immune function [73]. Changes in PC metabolism likely stem from tramadol-driven effects on monoaminergic systems and oxidative stress, with implications for membrane structure and cholinergic neurotransmission [74]. Altered lysine, methionine, cysteine, SAM, and taurine pathways suggest increased hepatic CYP450-mediated detoxification and redox adaptation in response to tramadol biotransformation [75]. Elevated acyl-glycine species indicate the disruption of mitochondrial fatty acid β-oxidation and enhanced glycine conjugation, pointing to altered energy metabolism and detoxification [76]. Collectively, these pathway perturbations implicate tramadol in broad neurochemical and metabolic reprogramming, with potential ramifications for mood, cognition, and metabolic health, warranting further investigation. Significant correlations were identified between various clinical parameters and metabolites. Most notably, the strong inverse correlation between HOMA-IR and carboxyethyl-GABA suggests a profound interplay between insulin resistance and GABAergic signaling. This finding is especially relevant given that GABA not only serves as a key neurotransmitter but also plays a critical role in pancreatic β-cell function, potentially influencing β-cell regeneration and insulin sensitivity [70,71]. Although carboxyethyl-GABA has not yet been established as a clinical biomarker, GABAergic metabolites are implicated in both metabolic and neuropsychiatric disorders and are modulated by the gut–brain axis [77,78,79]. Quinolinate is a recognized marker of neurotoxicity and neuroinflammation [80]. In our cohort, tramadol administration selectively altered both quinolinate and carboxyethyl-GABA, indicating targeted effects on the kynurenine and GABAergic pathways. Pathway enrichment demonstrated broader, yet non-global, disturbances in amino acid and lipid metabolism. These specific neuroactive metabolite shifts may contribute to interindividual variability in tramadol response, supporting quinolinate and carboxyethyl-GABA as candidates for further evaluation as clinically relevant biomarkers of tramadol-induced neurochemical changes.

A recent study by Jiang et al. integrated proteomic and metabolomic profiling to examine the effects of chronic tramadol exposure in mice, revealing 31 differentially expressed proteins and 34 differentially expressed metabolites in the serum of tramadol-treated versus control animals [63]. Metabolomics analysis revealed that differentially expressed metabolites were mainly involved in protein ingestion and absorption, fatty acid biosynthesis, steroid hormone biosynthesis, and bile secretion [63]. Several of the pathways and metabolites identified in the controlled animal model, including perturbations in amino acid metabolism and markers of kidney and neurological function, such as creatinine (1-methylguanidine) and quinolinic acid, closely parallel those observed in our cohort. This concordance supports the biological validity of our findings and highlights the broad metabolic impact of chronic tramadol exposure. Our identification of metabolites, like taurine and quinolinic acid, previously implicated in neurotoxicity and mood disorders, offers additional mechanistic insight into the effects of prolonged tramadol use, suggesting that its metabolic influence extends beyond direct pharmacological targets to neurochemical, inflammatory, and organ-specific pathways. While CYP2D6 variability and other genetic factors remain important modulators of individual response, our data underscores the value of combined genomic and metabolomic profiling for detecting both shared and population-specific disruptions. However, the clinical relevance of these molecular signatures, for risk prediction, patient stratification, or treatment guidance, remains to be established. Larger, prospective, and longitudinal studies are needed to validate these findings and assess their predictive performance and integration into precision opioid prescribing.

Limitations exist in the study. While our study effectively targeted metabolomic signatures linked to tramadol consumption, the findings should be regarded as preliminary due to the limited sample size and partial data on treatment duration and adherence. Although all analyses were adjusted for multiple comparisons using FDR to mitigate type I error, the risk of false negative findings (type II error) remains, particularly where sample size may restrict statistical power. Furthermore, we must acknowledge that the observed metabolomic changes could also be driven by external factors, such as diet, disease status, genetic predispositions, and other environmental influences. Therefore, further detailed investigations with larger, well-characterized cohorts and rigorous control for confounding variables are needed to clarify the specificity and clinical relevance of these metabolomic alterations. The molecular signatures identified in this study remain exploratory and hypothesis-generating; given our sample size and cross-sectional design, their predictive value or clinical utility for tramadol prescribing or patient stratification cannot yet be established. Further validation in independent, longitudinal cohorts will be necessary to confirm their significance and support clinical translation.

## 4. Materials and Methods

### 4.1. Data Source and Study Participants

The data for this study were obtained from participants enrolled in the QBB, a comprehensive repository comprising deep phenotyping information on both Qatari nationals and long-term residents (individuals residing in Qatar for ≥15 years) aged 18 years and older. However, for the purposes of this study, only data pertaining to Qatari participants were utilized. The QBB database provided extensive baseline socio-demographic information, clinical and behavioral phenotypic data, and various health metrics. These metrics included but were not limited to the body mass index, blood pressure, fasting glucose levels, insulin levels, lipid profile, liver and kidney enzymes, creatinine, citric acid, lactate, and a multitude of other clinical biochemistry parameters. All measurements were conducted at the central laboratory of Hamad Medical Corporation (HMC), which holds accreditation from the College of American Pathologists (CAP). Prior to participation, all individuals provided informed consent. In addition to clinical data, the QBB dataset incorporated responses to questionnaires that covered participants’ history of diabetes, medication usage, and metabolomics data for 1000 metabolites [81]. Subjects with a minimum of one of 3 tramadol metabolites present in the blood were selected as the study group. Controls were selected using propensity score matching using the R package (MatchIt) (V. 4.2.1) with the “nearest” method to minimize the discrepancy in terms of age, gender, and BMI. QBB data used in this study were limited to individuals recruited between 11 December 2012 and 9 June 2016, as no further metabolomic data have been generated or released by QBB since that period.

Within this encompassing dataset, prescription data for tramadol formulations (50 mg capsules, 100 mg SR tablets, and oral drops) were also extracted from Hamad Medical Corporation’s Cerner electronic health record system, spanning January 2019 to December 2022. This helped to examine prescription trends in relation to the analyzed data. By agreement with QBB and due to associated regulatory constraints, individual-level details concerning co-medication use, comorbidities, and precise duration or dosing history of tramadol exposure were not available for extraction and were therefore not included in the present analysis. All tramadol-positive participants had documented medications and reported clinical parameters within the QBB database; however, subgroup analyses based on co-medications or comorbid conditions were not feasible. This study was approved by the Institutional Review Boards (IRB) of QBB (QF-QBB-RES-ACC-00118) and HMC (MRC-01-22-088). In this study, data from 81 participants were analyzed to investigate the pharmacogenomic and pharmacometabolomic impacts of tramadol usage in the Qatari population. A specific group of 27 participants was identified with detectable tramadol metabolites; these individuals were actively taking tramadol with dosages ranging from 50 mg to a maximum of 400 mg, though dose-dependent effects were not specifically stratified in this analysis. The remaining 54 participants served as controls, including 24 individuals who were not on any medication. Participants positive for tramadol were compared to negative controls (included individuals negative for any illicit drugs), allowing for the identification of associated metabolic pathways and clinical traits.

### 4.2. Metabolomics

#### 4.2.1. Metabolomics Measurements

Blood plasma samples with ethylenediaminetetraacetic acid (EDTA) underwent analysis on the non-targeted metabolomics platform at Metabolon Inc. (Morrisville, NC, USA), conducted at the Anti-Doping Laboratory-Qatar (ADLQ), as previously outlined [82]. The analytical setup involved a Waters ACQUITY ultra-performance liquid chromatography (UPLC) (Waters Corporation, Milford, MA, USA) coupled with a Thermo Scientific Q-Exactive high-resolution/accurate mass spectrometer (Thermo Fisher Scientific, Inc., Waltham, MA, USA). This system utilized a heated electrospray ionization (HESI-II) source and an Orbitrap mass analyzer, operating at a resolution of 35,000 mass.

For comprehensive metabolite profiling, three analyses were performed using a C18 column, with one under acidic positive ion conditions and two under basic negative ion conditions, optimized for hydrophilic and hydrophobic compounds, respectively. An additional analysis was conducted using negative ionization following elution from a hydrophilic interaction liquid chromatography (HILIC) column. Metabolon’s proprietary software and a reference library containing over 3300 purified standard compounds facilitated the annotation of raw mass spectrometry (MS) data. Peak quantification was achieved through area-under-the-curve measurements. Normalization of data was executed in run-day blocks by aligning medians to equal one, ensuring proportional normalization of each data point. The overall relative standard deviation for instrument variability, as determined by internal standards, was 12%, and total process variability, based on endogenous metabolites in reference samples, was 16%. The dataset included a total of 1159 biochemicals, comprising 937 named biochemicals with known identities and 222 unidentified compounds (unknowns). Detailed information on the QBB metabolomics data can be found in Suhre et al. (2022) [82].

#### 4.2.2. Statistical Analysis

Metabolomics data underwent inverse rank normalization. Analysis was conducted using SIMCA^®^ software (Version 16.0.1), a powerful tool for multivariate data analysis employing advanced algorithms and interactive visualizations. Multivariate analyses included unsupervised methods, such as principal component analysis (PCA), and supervised methods, like orthogonal partial least square-discriminant analysis (OPLS-DA). Linear models for each metabolite were performed using R software (version 4.2.1), adjusting for confounders, such as age, gender, and BMI, and principal components 1 and 2 from PCA analysis. Nominal *p*-values underwent multiple testing correction using the false discovery rate (FDR), with statistical significance defined as FDR < 0.05. To gain insights into the functional implications of metabolomic alterations associated with tramadol use, a functional enrichment analysis was performed. This analysis utilized a one-way Wilcoxon rank sum test followed by FDR correction. Functional enrichment analysis was also run on all *p*-value ordered metabolite lists generated from a linear model in the study based on Fisher’s exact test, which was followed by FDR correction. Subpathways, previously defined by Metabolon using Creative Proteomics’ technology and sophisticated bioinformatics tools, were considered for enrichment analysis. Subpathways with less than three top hits were excluded from further analysis. Spearman’s correlation analysis was conducted to evaluate the relationship between various clinical parameters and significant metabolites in individuals using tramadol.

### 4.3. Genomics Analysis

Methods for whole-genome sequencing (WGS) and genetic variant identification in this cohort are described in detail elsewhere [83,84]. Briefly, blood samples collected by the Qatar Biobank were sequenced using Illumina HiSeq X instruments (Illumina, San Diego, CA, USA) to a mean coverage of 30×, as previously reported.

For the pharmacogenomics analysis, CYP2D6 genotypes were determined from the WGS BAM file using Cyrius software (https://github.com/Illumina/Cyrius, accessed on 2 December 2024), and metabolizer status was assigned based on activity scores following Pharmvar and CPIC/PharmGKB (level 1A evidence) guidelines (https://www.pharmgkb.org/page/cyp2d6RefMaterials (accessed on 2 December 2024) and https://cpicpgx.org/guidelines/guideline-for-codeine-and-cyp2d6/ (accessed on 2 December 2024). On this basis, participants were classified as poor, intermediate, normal, or ultra-rapid metabolizers. In addition, selected variants in other pharmacogenes relevant to tramadol pharmacokinetics and pharmacodynamics, including CYP3A4 (alleles *1, *22, *15, *36; function inferred by DPWG), CYP2B6 (alleles *1, *2, *4, *5, *6, *11), OPRM1 (rs1799971, rs62436463), and ABCB1 (rs1045642, rs1128503, rs2032582), were analyzed. These genes are classified as PharmGKB level 3 informative markers for tramadol, representing loci with limited or emerging evidence for clinical actionability. Further details are provided in Supplementary Table S1. All phenotype predictions were based on genotype data, as no direct enzymatic activity measurements were performed.

## 5. Conclusions

**Figure 5:**
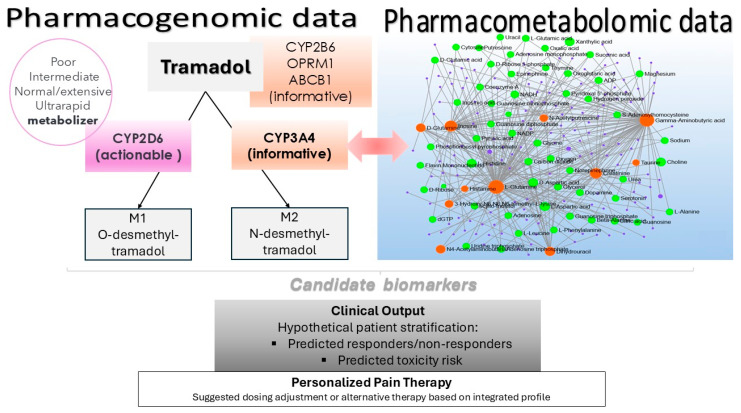
Integration of pharmacogenomic and pharmacometabolomic data for personalized tramadol therapy. The left panel summarizes key pharmacogenomic determinants of tramadol metabolism, including actionable (CYP2D6) and informative (CYP3A4) genes directly influencing the formation of the main tramadol metabolites, M1 and M2. Additional pharmacogenes (CYP2B6, OPRM1, ABCB1) are also shown for their potential relevance. The right panel depicts a metabolite interaction network generated from our experimental cohort data using MetaboAnalyst, highlighting metabolic perturbations associated with tramadol exposure. Integration of these datasets supports candidate biomarker discovery and patient stratification, providing a framework for individualized pain management.

Our study contributes to the understanding of tramadol’s metabolic impact and provides insights into possible therapeutic implications of identified pathways and biomarkers. Further validation in larger cohort studies will help enhance the clinical applicability of these findings. Additionally, considering the potential for personalized therapy based on individual genetic/metabolic profiles, as illustrated in Figure 5, should be a significant focus in future research aimed at optimizing treatment strategies for patients receiving tramadol. Despite inherent limitations, our data indicates that evaluating metabolomic changes with drug exposure could afford comprehensive insight into biochemical alterations caused by medications, like tramadol, which is invaluable information when aiming to minimize toxicity and maximize efficacy. This may lead not only to better monitoring methods for managing toxicities but also reveals potential mechanisms underlying observed differences in responses amongst individuals, thereby potentially enabling more effective individually tailored treatments in clinical practice.

## Figures and Tables

**Table 1:** Clinical characteristics of participants.

Variable	Tramadol (−)	Tramadol (+)	*p*-Value
*n*	54	27	
Diabetes status			0.482
Yes	8 (14.81%)	2 (7.41%)	
No	46 (85.19%)	25 (92.59%)	
Gender			
Male	18 (33.33%)	10 (37.04%)	0.934
Female	36 (66.67%)	17 (62.96%)	
Age	41.259 (12.407)	39 (13.89)	0.478
BMI	29.376 (5.563)	28.227 (5.819)	0.399
Average systolic BP	112.87 (14.721)	112.704 (12.892)	0.958
Average diastolic BP	74.963 (11.535)	73.148 (8.47)	0.425
Average pulse rate	70.574 (10.785)	72.593 (10.778)	0.431
Homa	2.445 (1.528–5.138)	2.08 (1.455–6.13)	0.845
Glucose (mmol/L)	5.1 (4.6–5.6)	5.1 (4.935–5.5)	0.821
Insulin (μU/mL)	11.25 (7.1–17)	10 (6.6–25.55)	0.837
HBA 1C	5.5 (5.3–5.8)	5.4 (5–5.6)	0.130
C Peptide (ng/mL)	2.68 (1.912–3.808)	2.61 (1.67–3.607)	0.731
Sitting height	127.2 (86.1–135.8)	87.3 (84.95–110.3)	0.052
Weight	78.048 (16.846)	74.089 (16.678)	0.320
Waist size	89.019 (14.423)	86.333 (16.117)	0.468
Hip size	108.13 (9.695)	104.259 (11.651)	0.144
Waist to hip ratio	0.822 (0.102)	0.825 (0.102)	0.903
Left handgrip	23 (20–34)	24 (20–35)	0.876
Right handgrip	30.204 (11.21)	31.231 (10.558)	0.691
Albumin (g/L)	44.907 (2.87)	44.963 (2.361)	0.926
Alkaline phosphatase (U/L)	66 (54.5–82.25)	63 (55.5–82.5)	0.992
ALT (GPT) (U/L)	18 (13–27.5)	18 (13.5–21.5)	0.688
AST GOT (U/L)	17 (15–20.75)	18 (13.5–21)	0.718
Bilirubin total (μmol/L)	6 (4.85–8.25)	6 (4.95–7.25)	0.963
GGT (U/L)	21 (13–30.5)	13.5 (12–24)	0.197
GGT-2 (U/L)	19 (14–30)	24 (14–37)	0.663
Bicarbonate (mmol/L)	26.093 (2.113)	26.741 (1.953)	0.176
Calcium (mmol/L)	2.36 (2.292–2.42)	2.41 (2.34–2.43)	0.091
Calcium corrected (mmol/L)	2.26 (2.2–2.31)	2.3 (2.25–2.325)	0.083
Chloride (mmol/L)	101.389 (2.422)	101.185 (2.512)	0.729
Creatine kinase (μ/L)	75 (56–118)	83.5 (45–130.25)	0.814
Creatine kinase_1 (ng/mL)	1.15 (0.76–1.73)	1.43 (1.15–1.71)	0.927
Creatine kinase_2 (U/L)	69 (55–83.5)	64.5 (63.75–65.25)	0.884
Creatinine (μmol/L)	64.796 (13.273)	67.556 (14.148)	0.402
Total protein (g/L)	72.889 (3.694)	73.593 (3.238)	0.383
Phosphorus (mmol/L)	1.104 (0.165)	1.108 (0.177)	0.914
Potassium (mmol/L)	4.369 (0.367)	4.333 (0.385)	0.696
Homocysteine (μmol/L)	8.5 (7.025–9.475)	7.8 (6.55–8.675)	0.079
Magnesium (mmol/L)	0.834 (0.055)	0.834 (0.064)	0.969
Sodium (mmol/L)	140 (139–141)	140 (139–141.5)	0.819
Urea (mmol/L)	4.25 (3.5–4.825)	3.7 (3.35–4.7)	0.637
Uric acid (μmol/L)	297.407 (73.841)	285.407 (93.336)	0.563
C reactive protein (mg/L)	5 (5–5.25)	5 (5–8)	0.200
Hematocrit (%)	40.207 (4.622)	40.456 (4.243)	0.811
Hemoglobin (g/dL)	13.2 (1.689)	13.374 (1.593)	0.653
Hemoglobin estimated from measured HCT	17.148 (3.509)	16.478 (3.391)	0.595
Basophil auto (%)	0.7 (0.5–0.8)	0.7 (0.45–0.8)	0.818
Basophils auto (×10^3^ μL)	0 (0–0.1)	0 (0–0.06)	0.407
Eosinophil auto (%)	2.5 (1.425–3.875)	3.2 (1.85–4.2)	0.205
Eosinophils auto (×10^3^ μL)	0.15 (0.1–0.275)	0.2 (0.1–0.3)	0.404
Lymphocyte auto (×10^3^ μL)	2 (1.9–2.475)	2.2 (1.7–2.6)	0.928
Lymphocyte auto (%)	34.183 (7.02)	37.078 (10.802)	0.214
Mean cell hemoglobin (pg)	27.5 (25.625–29.2)	27.9 (26.4–29.15)	0.458
Mean cell hemoglobin concentration (g/dL)	32.88 (0.965)	33.052 (1.171)	0.513
Mean cell volume (fl)	83.25 (78.35–87.8)	84.7 (80.4–87.6)	0.558
Mean platelet volume (fl)	9.104 (1.074)	9.481 (1.037)	0.133
Monocyte auto (%)	7.15 (5.6–8.775)	7.6 (6.5–9.8)	0.290
Monocytes auto (×10^3^ μL)	0.45 (0.4–0.5)	0.4 (0.4–0.6)	0.984
Neutrophil auto (×10^3^ μL)	3.6 (3–4.3)	3 (2.3–3.65)	0.060
Neutrophil auto (%)	55.067 (8.329)	51.052 (10.87)	0.099
Platelet (×10^3^ uL)	247.868 (48.249)	243.889 (71.005)	0.795
Red blood cell (×10^6^ μL)	4.907 (0.611)	4.848 (0.454)	0.625
White blood cell (×10^3^ μL)	6.4 (5.525–7.5)	5.7 (5.2–6.55)	0.096
RDW	13.9 (13.325–15.275)	13.9 (13.25–14.75)	0.616
Iron (μmol/L)	15.449 (5.896)	15.124 (5.72)	0.812
Total iron binding capacity (μmol/L)	59.5 (54–63.75)	57 (55–62)	0.710
UIBC (μmol/L)	43 (35.15–50)	41.2 (36.6–50.1)	0.924
Ferritin (μg/L)	39.5 (18.5–103.5)	29 (12–96)	0.620
Cholesterol total (mmol/L)	4.966 (0.922)	4.949 (1.061)	0.943
HDL cholesterol (mmol/L)	1.195 (1.062–1.485)	1.33 (1.08–1.585)	0.455
LDL cholesterol calc (mmol/L)	2.998 (0.838)	2.979 (0.934)	0.932
Triglyceride (mmol/L)	1.22 (0.957–1.895)	1.06 (0.705–1.85)	0.198
Thyroid-stimulating hormone (mIU/L)	1.42 (0.952–2.175)	1.36 (1.05–2.125)	0.900
Free thyroxine (pmol/L)	12.7 (12.125–14.687)	12.76 (11.555–14.845)	0.768
Free triiodothyronine (pmol/L)	4.421 (0.629)	4.298 (0.566)	0.394
Sex hormone binding globulin (nmol/L)	44.95 (32.65–60.75)	41.1 (32.25–56.5)	0.544
Estradiol (pmol/L)	127 (76–318)	112.5 (68.25–356.75)	0.929
Testosterone total (nmol/L)	1.54 (0.88–10.91)	1.71 (0.863–11.835)	0.900
Vitamin B12 (pmol/L)	253.5 (194.5–314.5)	277 (223–342.5)	0.213
Folate (nmol/L)	25.05 (19.225–29.95)	25.35 (22.325–30.625)	0.583
Dihydroxyvitamin D total (ng/mL)	17.5 (13–24)	16 (12–21)	0.648
Myoglobin (ng/mL)	21 (21–21.75)	21 (20–25)	0.937
NT proBNP (pg mL)	27 (12.86–43.25)	21.3 (10–37.65)	0.376
International normalization ratio	1 (1–1.1)	1.1 (1–1.1)	0.040
Activated partial thromboplastin time (seconds)	33.8 (32–36.175)	34.7 (32.85–36.85)	0.247
Prothrombin time PT (seconds)	11.05 (10.625–11.875)	11.8 (10.9–12.7)	0.032
Rheumatoid factor (IU/mL)	10 (9.825–10)	10.35 (9.875–11.1)	0.280
Fibrinogen (g/L)	3.17 (2.725–3.7)	3.1 (2.885–3.55)	0.984

**Table 2:** Results from linear regression analysis show the top FDR significant metabolites associated with tramadol-positive individuals.

Metabolite	Superpathway	Subpathway	Estimate	SE	*p*-Value	FDR
1-methylguanidine	Amino Acid	Guanidino and Acetamido Metabolism	1.752	0.221	5.10 × 10^−11^	4.16 × 10^−8^
quinolinate	Cofactors and Vitamins	Nicotinate and Nicotinamide Metabolism	1.268	0.178	5.80 × 10^−10^	2.15 × 10^−7^
*N*2,*N*2-dimethylguanosine	Nucleotide	Purine Metabolism, Guanine Containing	1.266	0.179	7.91 × 10^−10^	2.15 × 10^−7^
*N*1-methylinosine	Nucleotide	Purine Metabolism, (Hypo)Xanthine/Inosine Containing	1.247	0.192	9.06 × 10^−9^	1.85 × 10^−6^
1-methyl-5-imidazoleacetate	Amino Acid	Histidine Metabolism	1.326	0.207	1.30 × 10^−8^	2.12 × 10^−6^
1-methyl-4-imidazoleacetate	Amino Acid	Histidine Metabolism	1.008	0.174	1.52 × 10^−7^	2.07 × 10^−5^
4-acetamidobutanoate	Amino Acid	Polyamine Metabolism	1.138	0.203	3.56 × 10^−7^	3.76 × 10^−5^
hydantoin-5-propionate	Amino Acid	Histidine Metabolism	1.384	0.238	3.68 × 10^−7^	3.76 × 10^−5^
3-phosphoglycerate	Carbohydrate	Glycolysis, Gluconeogenesis, and Pyruvate Metabolism	1.030	0.198	1.83 × 10^−6^	1.50 × 10^−4^
creatinine	Amino Acid	Creatine Metabolism	0.836	0.173	6.79 × 10^−6^	5.04 × 10^−4^
*N*-acetyl-isoputreanine	Amino Acid	Polyamine Metabolism	0.934	0.197	1.03 × 10^−5^	6.97 × 10^−4^
glutamine conjugate of C6H10O2 (1)*	Partially Characterized Molecules	Partially Characterized Molecules	0.882	0.186	1.28 × 10^−5^	8.02 × 10^−4^
hydroxy-*N*6,*N*6,*N*6-trimethyllysine*	Amino Acid	Lysine Metabolism	0.741	0.167	3.21 × 10^−5^	1.87 × 10^−3^
1-methylurate	Xenobiotics	Xanthine Metabolism	0.902	0.205	3.73 × 10^−5^	2.03 × 10^−3^
5,6-dihydrouracil	Nucleotide	Pyrimidine Metabolism, Uracil Containing	−0.974	0.243	1.76 × 10^−4^	8.65 × 10^−3^
1-ribosyl-imidazoleacetate*	Amino Acid	Histidine Metabolism	0.675	0.171	1.80 × 10^−4^	8.65 × 10^−3^
carboxyethyl-GABA	Amino Acid	Glutamate Metabolism	0.843	0.226	3.77 × 10^−4^	1.71 × 10^−2^
glutarylcarnitine (C5-DC)	Amino Acid	Lysine Metabolism	0.789	0.212	4.45 × 10^−4^	1.91 × 10^−2^
taurine	Amino Acid	Methionine, Cysteine, SAM and Taurine Metabolism	0.775	0.223	8.57 × 10^−4^	3.50 × 10^−2^
phosphoethanolamine	Lipid	Phospholipid Metabolism	0.736	0.216	1.06 × 10^−3^	4.14 × 10^−2^
5-(galactosylhydroxy)-L-lysine	Amino Acid	Lysine Metabolism	0.788	0.235	1.27 × 10^−3^	4.57 × 10^−2^
cysteine sulfinic acid	Amino Acid	Methionine, Cysteine, SAM and Taurine Metabolism	0.751	0.223	1.29 × 10^−3^	4.57 × 10^−2^
*N*-acetyl-1-methylhistidine*	Amino Acid	Histidine Metabolism	0.590	0.178	1.43 × 10^−3^	4.88 × 10^−2^

Asterisk (*) next to a metabolite indicates a compound that has not been officially confirmed based on a reference standard, but Metabolon is confident in its identity.

**Table 3:** Results from functional enrichment analysis using Fisher’s exact test.

Subpathways	*p*-Value	FDR
Histidine Metabolism	0.000178	0.018
Fatty Acid Metabolism (Acyl Glycine)	0.011	0.499
Methionine, Cysteine, SAM and Taurine Metabolism	0.015	0.499
Phosphatidylcholine (PC)	0.028	0.692
Lysine Metabolism	0.035	0.700

## Data Availability

The data that have been used are confidential.

## Supplementary Materials

The following supporting information can be downloaded at: https://www.mdpi.com/article/10.3390/ph18070971/s1, Figure S1: Capsules, 100 mg sustained-release tramadol tablets, and 100 mg/mL (10 mL) tramadol oral drops. Tramadol prescribing patterns in Qatar over a four-year period. The figure shows monthly prescription counts for 50 mg tramadol capsules, 100 mg sustained-release tramadol tablets, and 100 mg/mL (10 mL) tramadol oral drops. A total of 4712 patients and 12,319 prescriptions were included during the study period (January 2019–December 2022). The *x*-axis indicates months, and the *y*-axis indicates the number of prescriptions per formulation per month. Figure S2: Tramadol prescribing trends by pain indication in Qatar over a four-year period. The figure shows monthly prescription counts for tramadol in the management of acute pain, cancer-associated pain, chronic pain, neuropathic pain, and psychogenic pain. A total of 4712 patients and 12,319 prescriptions were included from January 2019 to December 2022. The *x*-axis indicates months, and the *y*-axis indicates the number of prescriptions per pain indication per month. Figure S3: Metabolome view map of significant metabolic pathways characterized in our study in response to tramadol. The map generated using MetaboAnalyst, displaying the statistical significance (−log10 *p*-value, *y*-axis) and pathway impact score (*x*-axis) for each affected metabolic pathway. Bubble size reflects the pathway impact; color indicates significance (yellow: lower; red: higher). Key altered pathways, such as phosphatidylcholine, histidine, and lysine metabolism, are shown, reflecting the principal findings of our analysis. Table S1: Pharmacogenetic variant summary. Table S2: Average concentrations of tramadol and O-desmethyltramadol_glucuronide, across different CYP2D6 metabolizer phenotypes. Table S3: Tramadol metabolism and CYP2D6 activity profiles in study participants. Table S4: Correlation coefficient. Table S5: Correlation *p*-value.

## Abbreviations

The following abbreviations are used in this manuscript:

QBB	Qatar Biobank
HMC	Hamad Medical Corporation
PGx	Pharmacogenomics
PMx	Pharmacometabolomics
EMR	Electronic medical records
PMs	Poor metabolizers
IMs	Intermediate metabolizers
NMs	Normal metabolizers
UMs	Ultra-rapid metabolizers
GGT	Gamma-glutamyl transferase
