# Supplementary material for: Pharmacogenomics and Pharmacometabolomics in Precision Tramadol Prescribing for Enhanced Pain Management: Evidence from QBB and EMR Data
Source: Pharmaceuticals (Basel). 2025 Jun 27;18(7):971. doi: 10.3390/ph18070971 (PMC12300021; doi:10.3390/ph18070971)
Supplement: Supplementary file 1 [file pharmaceuticals-18-00971-s001.zip › Tramadol Suppl.pdf]

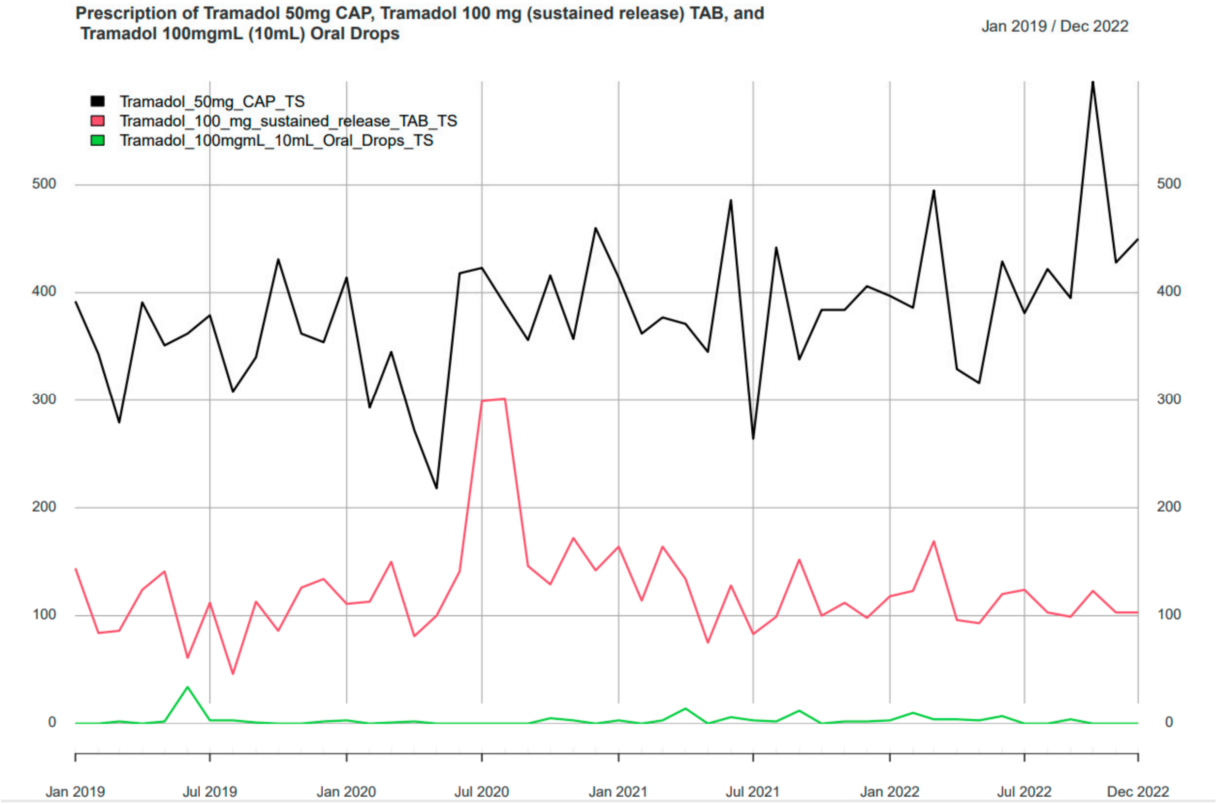

Figure S1. Tramadol prescribing patterns in Qatar over a four-year period. The figure shows monthly prescription counts for Tramadol 50 mg capsules, Tramadol 100 mg sustained-release tablets, and Tramadol 100 mg/mL (10 mL) oral drops. A total of 4,712 patients and 12,319 prescriptions were included during the study period (January 2019 – December 2022). The *x*-axis indicates months, and the y-axis indicates the number of prescriptions per formulation per month.

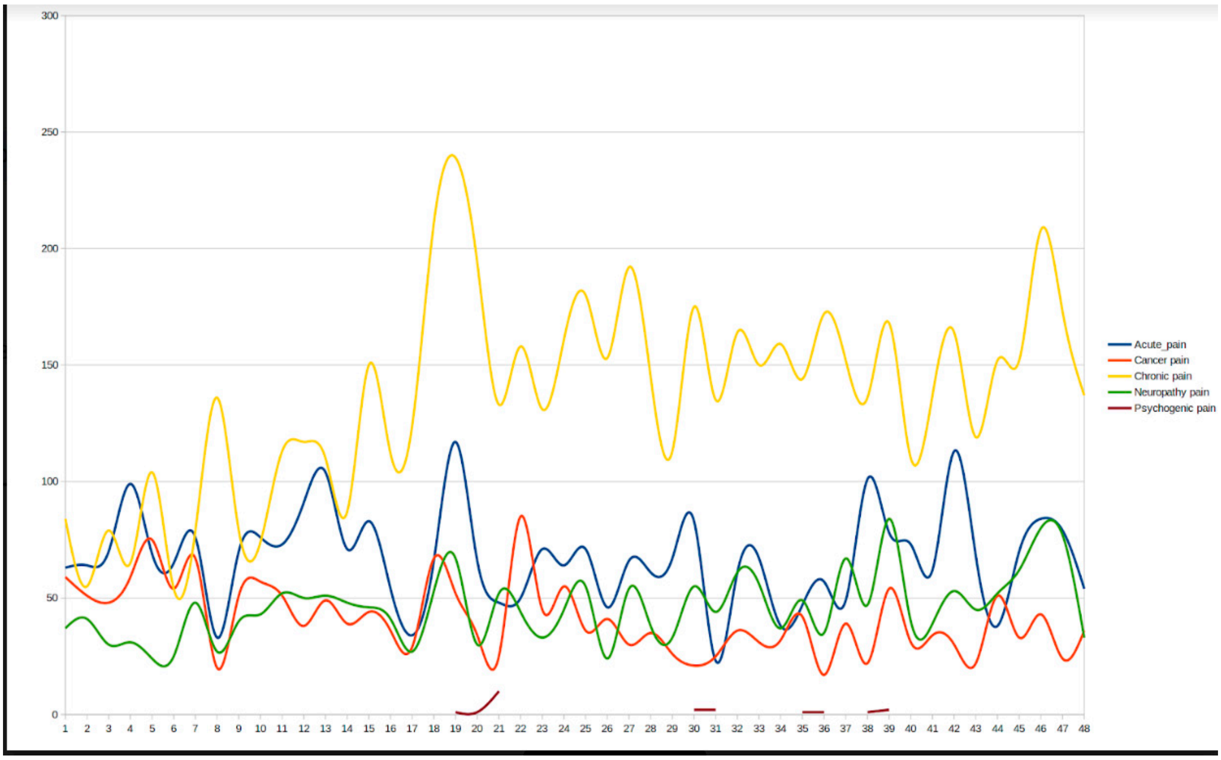

Figure S2. Tramadol prescribing trends by pain indication in Qatar over a four-year period. The figure shows monthly prescription counts for tramadol in the management of acute pain, cancer-associated pain, chronic pain, neuropathic pain, and psychogenic pain. A total of 4,712 patients and 12,319 prescriptions were included from January 2019 to December 2022. The *x*-axis indicates months, and the *y*-axis indicates the number of prescriptions per pain indication per month.

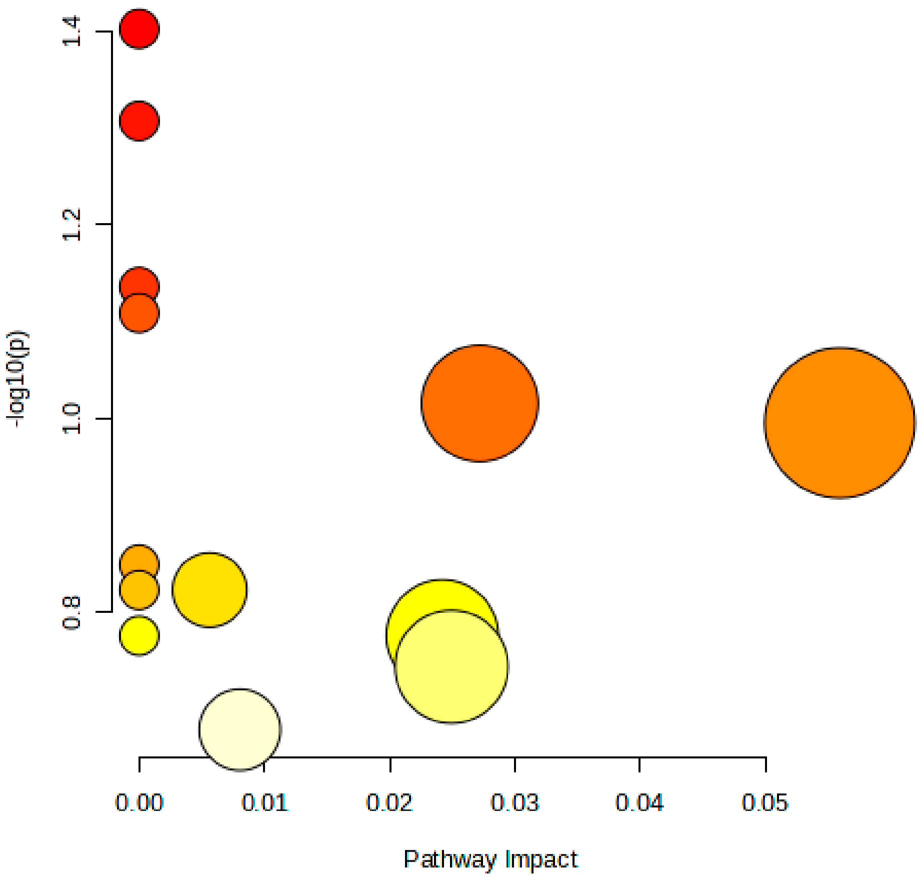

Figure S3. Metabolome view map of significant metabolic pathways characterized in our study in response to tramadol. The map generated using MetaboAnalyst, displaying the statistical significance (−log10 *p*-value, *y*-axis) and pathway impact score (*x*-axis) for each affected metabolic pathway. Bubble size reflects pathway impact; color indicates significance (yellow: lower; red: higher). Key altered pathways such as phosphatidylcholine, histidine, and lysine metabolism are shown, reflecting the principal findings of our analysis.

Table S2. Average Concentrations of Tramadol and O-desmethyltramadol_glucuronide, across Different CYP2D6 Metabolizer Phenotypes

	**Tramadol**	**O-desmethyltramadol_glucuronide**
**Poor**	1.3	1.26
**Intermediate**	0.97	1.004
**Normal**	0.94	0.78
**Ultra-rapid**	0	1

Table S3. Tramadol Metabolism and CYP2D6 Activity Profiles in Study Participants

my_ID	Tramadol	O-desmethyltramadol_glucuronide	CYP2D6_activity_scores	Metabolizer_status
**PT1**	1.3208	1.2672	0	Poor
**PT2**	1.5013	2.1781	0.5	Intermediate
**PT3**	0.9693	1.2409	0.5	Intermediate
**PT4**	1.5265	1.4248	1	Intermediate
**PT5**	1	0	1	Intermediate
**PT6**	1	1	1	Intermediate
**PT7**	0.9866	1.3177	1	Intermediate
**PT8**	0.8309	0.875	1	Intermediate
**PT9**	0.0064	0	1	Intermediate
**PT10**	NA	1	1.25	Normal
**PT11**	0.9789	0.7829	1.25	Normal
**PT12**	0.8635	0.7339	1.25	Normal
**PT13**	0.7048	0.6698	1.25	Normal
**PT14**	1.0638	1	1.5	Normal
**PT15**	0.9767	1.3655	1.5	Normal
**PT16**	0.6121	0	1.5	Normal
**PT17**	1.8037	1.1009	2	Normal
**PT18**	1.6317	1.6917	2	Normal
**PT19**	1.2398	0	2	Normal
**PT20**	1.1669	0.9546	2	Normal
**PT21**	1	0	2	Normal
**PT22**	0.9978	0.6265	2	Normal
**PT23**	0.9943	0.9909	2	Normal
**PT24**	0.6813	0.7378	2	Normal
**PT25**	0.53	0.6785	2	Normal
**PT26**	0.8781	0.9909	2.25	Normal
**PT27**	NA	1	3	Ultra-rapid
